# Virus Association with Bacteria and Bacterial Cell Components Enhance Virus Infectivity

**DOI:** 10.1007/s12560-025-09633-7

**Published:** 2025-01-09

**Authors:** Wenjun Deng, Giselle Almeida, Kristen E. Gibson

**Affiliations:** 1 Present Address: College of Life Science, Qingdao University, Qingdao, People’s Republic of China; 2 Division of Agriculture, Department of Food Science, University of Arkansas, 1371 West Altheimer Dr, Fayetteville, AR 72704 USA; 3 Present Address: Arkansas Children’s Hospital, Little Rock, AR USA

**Keywords:** Human norovirus, Tulane virus, Murine norovirus, Aichi virus, *Bacillus cereus*, *Enterobacter cloacae*

## Abstract

The transmission and infection of enteric viruses can be influenced by co-existing bacteria within the environment and host. However, the viral binding ligands on bacteria and the underlying interaction mechanisms remain unclear. This study characterized the association of norovirus surrogate Tulane virus (TuV) and murine norovirus (MNV) as well as the human enteric virus Aichi virus (AiV) with six bacteria strains (*Pantoea agglomerans*, *Pantoea ananatis*, *Bacillus cereus*, *Enterobacter cloacae*, *Exiguobacterium sibiricum*, *Pseudomonas* spp.). At room temperature, the viruses bound to all bacteria in strain-dependent rates and remained bound for at least 2 h. The virus association with two gram-positive bacteria *B. cereus* and *E. sibiricum* was less efficient than gram-negative bacteria. Next, the bacterial envelope components including lipopolysaccharides (LPS), extracellular polymeric substances (EPS), and peptidoglycan (PG) from selected strains were co-incubated with viruses to evaluate their effect on virus infectivity. All the tested bacteria components significantly increased virus infection to variable degrees as compared to PBS. The LPS of *E. coli* O111:B4 resulted in the greatest increases of infection by 0.19 log PFU for TuV as determined by plaque assay. Lastly, bacterial whole cell lysate of *B. cereus* and *E. cloacae* was examined for their impact on the infectivity of MNV and TuV. The co-incubation with whole cell lysates significantly increased the infectivity of TuV by 0.2 log PFU but not MNV. This study indicated that both the individual bacteria components and whole bacterial cell lysate can enhance virus infectivity, providing key insights for understanding virus–bacteria interaction.

## Introduction

Enteric viruses are a group of viruses that infect and replicate in the gastrointestinal tract of its specific host, leading to symptoms such as diarrhea and vomiting (Fong & Lipp, 2005; Oude Munnink & van der Hoek, 2016). These viruses are transmitted through fecal–oral route, including contaminated food or water and person-to-person contact (Gómez-Mascaraque et al., 2018). The most common foodborne enteric viruses include human norovirus (HuNoV), human rotavirus, human astrovirus, human adenovirus, human sapovirus, and human Aichi virus (AiV) (Oude Munnink & van der Hoek, 2016). In particular, HuNoV was responsible for 18% of global diarrhea cases, with similar prevalences across all age groups (Lopman et al., 2016). From 1990 to 2019, the age-standardized death rate—a weighted age-specific mortality rate—caused by HuNoV has decreased from 5.02 to 1.86 per million people worldwide (Zhang et al., 2022). However, this rate increased for developed countries, primarily attributed to the higher death rates in elderly. In the food continuum, over 50% of foodborne outbreaks were caused by HuNoV, either via contamination by food handlers or direct food contamination during production (U.S. Centers for Disease Control, 2024).

Human noroviruses are non-enveloped single-strand positive sense RNA viruses belonging to the *Caliciviridae* family (Karst, 2010). Until recently, HuNoV was unable to be propagated in a cell culture system (Ettayebi et al., 2016; Jones et al., 2014) or replicated using an animal model (Dycke et al., 2019; Tan et al., 2023). Due to the lack of animal cell lines for HuNoV cultivation, surrogate viruses such as Tulane virus (TuV) and murine noroviruses (MNV) have been studied to help understand the biological mechanism of HuNoV (Baldridge et al., 2015; Drouaz et al., 2015). MNV shares genetic and structural similarities with HuNoV. It is the first norovirus that could be replicated in cell culture as well as a small animal model (Hirneisen & Kniel, 2013; Wobus et al., 2006). Though TuV belongs to a different genus *Recovirus*, sequencing has shown its close relation to genogroup II HuNoV (Farkas et al., 2014). Unlike MNV which exhibits limited genetic variability, *Recovirus* has a large biological diversity similar to HuNoV (Farkas et al., 2014). In fact, the virus–bacteria associations discussed below have also been observed not only in HuNoV but surrogate viruses as well. A study on MNV in mice revealed that gut bacterial components are required for persistent infection of viruses (Baldridge et al., 2015). In addition, TuV can recognize histo-blood group antigens (HBGA) and were reported to bind to HBGA-expressing bacteria although with slightly lower binding efficiency than HuNoV (Almand et al., 2017).

Over the past decade, studies have been reported on the promotive or inhibitory roles that commensal bacteria play in the replication, transmission, recombination, infection, and persistence of enteric viruses (Erickson et al., 2018; Kuss et al., 2011; Lian et al., 2022; Neu & Mainou, 2020; Robinson et al., 2014; Shi et al., 2019). Kuss et al. (2011) observed that the infection and replication of poliovirus were reduced in antibiotic-treated mice intestine where microbiota were depleted. Moreover, the exposure of poliovirus to N-acetylglucosamine-containing polysaccharides on the surfaces of gut bacteria increased virus infectivity both in vivo and in vitro. Similarly, HuNoV has also been reported to be associated with enteric bacteria. Miura et al. (2013) observed the binding of HuNoV-like particles (HuNoVLP) to the enteric bacteria *Enterobacter* sp. SENG-6 and further revealed that the binding was mainly due to the HBGA-like compounds present in the extracellular polymeric substances (EPS) of bacteria. This is particularly relevant as HBGAs located on cells that line the human intestinal tract are the presumed receptor for the majority of HuNoV genotypes including the predominant genotype (GII.4) associated with human infections (Farahmand et al., 2022; Ruvoën-Clouet et al., 2014; Zhang et al., 2024). Similar findings were reported in the study by Li et al. (2015) who observed the binding of HuNoVLPs to eleven HBGA-expressing bacteria. The binding to bacteria protected HuNoVLPs under acute heat stress by maintaining the antigen integrity and mucin binding ability of the VLP. Almand et al. (2017) characterized the binding efficiency of HuNoV to gut microbiota and lab reference strains. HuNoV showed high binding efficiency (< 10% unbound) to all tested bacteria, although the binding types were not specified in the study.

Given the findings on the associations between enteric viruses and bacteria, more investigations are needed to understand the role of bacteria in the infection cycle of enteric viruses, specifically HuNoV. The research on HuNoV and enteric bacteria interactions are limited, and the impact of bacterial cell components on viruses remain poorly understood. While previously Miura et al. (2013) reported stable binding between HuNoVLP and bacterial EPS, Almand et al. (2017) observed VLP binding to bacteria membrane and pili but found no consistent binding to EPS. In addition, recent studies revealed that the interaction between MNV and outer membrane vesicles of commensal bacteria reduced the viral infectivity in macrophages and that MNV can alter the expression of bacterial vesicle components (Bhar et al., 2022; Mosby et al., 2022). These studies suggested complex and diverse ways of virus and bacteria interaction that need to be further explored.

As many questions regarding virus–bacteria associations remain, the aim of this study was to characterize the virus–bacteria association at room temperature (RT) for two HuNoV surrogates, TuV and MNV, as well as for AiV—a human pathogenic enteric virus. Room temperature was selected as it represents a range of real-world conditions that enteric viruses could persist and transmit, including food service environments (e.g., food handling and preparation), surfaces in private homes and public areas, as well as person-to-person contact (de Graaf et al., 2017; Derrick et al., 2021). The associations between virus and whole cell bacteria were observed over time at RT. Additionally, the infectivity of viruses was evaluated after incubation with lysed bacterial cells and specific bacterial cell envelope components (BEC), including LPS, EPS, and peptidoglycan (PG).

## Materials and Methods

### Virus Propagation and Titration

Tulane virus (TuV), obtained courtesy of Dr. Jason Jiang (Cincinnati Children’s Hospital Medical Center; Cincinnati, OH), was cultivated in LLC-MK2 cell line [ATCC CCL-7; American Type Culture Collection (ATCC), Manassas, VA]. Murine norovirus type 1 (MNV-1), obtained courtesy of Dr. Kellogg Schwab (Johns Hopkins Bloomberg School of Public Health; Baltimore, MD), was propagated in RAW 264.7 (ATCC TIB-71). Aichi virus (AiV) was kindly provided by Dr. Pierre Pothier at Dijon University Hospital in Dijon, France, and AiV were cultivated in Vero cells (ATCC CCL-81). Propagation and titration of all viruses were performed as described previously by Deng et al., (2019). Briefly, host cells were infected by viruses at MOI 0.1 and incubated for 48–72 h. For virus harvest, viruses were released from the infected cells by applying three cycles of freezing–thawing at − 80 °C and separated from the cell debris by centrifugation at 4 °C. Virus cell lysates were then purified by filtration through a 0.22-µm mixed cellulose ester membrane (Millex-GS; MilliporeSigma, Burlington, MA), aliquoted, and stored at − 80 °C. Stocks of TuV and MNV-1 were further concentrated by ultracentrifugation at 130,000×*g* for 1 h (Optima™ MAX-XP Beckman Coulter; Indianapolis, IN) and stored at − 80 °C.

### Bacteria Culture and Isolation of Bacterial Envelope Components

Four lab bacteria strains including *Pantoea agglomerans* ATCC 27155, *Pantoea ananatis* ATCC 27996, *Bacillus cereus* ATCC 14579, and *Enterobacter cloacae* ATCC 13047, as well as two bacteria strains, *Exiguobacterium sibiricum* and *Pseudomonas* spp., isolated from lettuce were used in this study. Bacteria were cultured aerobically in 500-mL flasks containing 250 mL of Nutrient Broth (NB) (Difco, Sparks, MD) under 30 °C and 160 rpm for 24 h. Three bacterial envelope components (BECs)—exopolysaccharides (EPS), lipopolysaccharide (LPS), and peptidoglycan (PG)—were extracted from selected bacteria following methods described as below.

#### Exopolysaccharides (EPS)

Exopolysaccharides were extracted from *B. cereus*, *E. cloacae*, and *P. ananatis*, respectively*.* The isolation procedures of EPS were based on the work by Miura et al. (2013). Briefly, the cultured cells (10^9^ CFU/mL) were centrifuged at 3000×*g* for 5 min. The cell pellet was then resuspended in phosphate-buffered saline (PBS) by vortexing. Afterward, the suspension was centrifuged again at 9000×*g* for 5 min. The supernatant was subsequently filtered with a 0.22-µm mixed cellulose ester membrane (Millex-GS; MilliporeSigma) and purified by a regenerated cellulose dialysis tubing (molecular weight cut-off of 3.5–14 kDa) (Fisher Scientific, Pittsburgh, PA). Then, 15 mL of purified solution was lyophilized at − 74 °C (Model 25XL; VirTis Company, Gardiner, NY) using a 50 -L bio-reaction tube and then stored at − 20 °C.

#### Lipopolysaccharides (LPS)

*P. ananatis* and *E. cloacae* culture adjusted to OD_600_ 0.9 were subjected to LPS extraction using a LPS extraction kit (iNtRON Biotechnology, Inc., Kyunggi, Korea) according to the manufacturer’s instructions with modifications (Lee & Baek, 2013). Following LPS extraction, the LPS pellet was dissolved via incubation with 5 µL of Proteinase K solution (13 mg/mL) for 30 min at 50 °C followed by centrifugation at 13,000×*g* for 3 min. The resulting LPS pellet was resuspended in 100-µL endotoxin-free distilled water. To establish a standard curve, the endotoxin units of LPS were quantified using a Chromogenic Endotoxin Quantitation kit (Pierce™ LAL, Rockford, IL) following the manufacturer’s protocol, and the absorbance was measured at OD_405_ (Microplate reader 3550; BIO-RAD, Hercules, CA).

#### Peptidoglycan (PG)

The extraction of PG was based on aqueous phenol extraction (v/v) methodology described by Ziamko and Okulich (2014) with modifications. Briefly, *B. cereus* overnight culture in NB was utilized as opposed to on meat-peptone agar. Preparation of bacterial culture after pelleting and extraction of PG were performed as described previously (Ziamko & Okulich, 2014). For confirmation, the PG isolated from *B. cereus* as well as a positive control stock of PG (*Bacillus subtilis*-TLR2 ligand; InvivoGen, San Diego, CA) were labeled with 0.5% Congo red solution at a 1:2 vol/vol ratio for 20 min. Excess stain was removed by centrifugation of samples at 1000 rpm for 75 min followed by washing with 0.5 mL 0.9% saline solution. These steps were repeated two times with a final centrifugation and storage of stained PG pellets at 4 °C. Stained PG pellets were resuspended in 100-µL endotoxin-free distilled water and visualized by confocal laser scanning microscopy (Leica TCS SP5 II; Leica Microsystems, Wetzlar, Germany).

#### Ultrasonic Lysing of Bacterial Cells

*B. cereus* and *E. cloacae* were selected as representatives for gram-positive and gram-negative bacteria, respectively. Each bacterium was grown in 10 mL of NB overnight at 30 °C and 200 rpm. Then, 1 mL of each culture at OD_600_ = 1 were pelleted at 8000×*g* for 5 min and washed twice with 0.1-M PBS. The resuspended pellet was exposed to sonication (Misonix, Inc., Farmingdale, NY) for 5 cycles over 5 min or 6 cycles over 7 min for *E. cloacae* and *B. cereus*, respectively, depending on the gram type of bacteria. Bacterial cell lysates were stored at − 20 °C.

### Virus–Bacteria Binding Assays

#### Whole Cell Bacteria and Virus

Experiments were carried out based on the method described by Miura et al. (2013) with modifications. Bacteria were cultured in 10-mL NB at 30 °C and 200 rpm for 24 h. The bacteria were pelleted by centrifugation at 8000×*g* for 10 min and resuspended in 10-mL PBS. The cell suspension was adjusted to a concentration of 10^9^ CFU/mL. One milliliter of each bacteria suspension was washed twice by centrifugation at 3000×*g* for 5 min with PBS and then mixed with 400 µL of each virus individually, including TuV (approximately 6.5 log PFU), AiV (approximately 7.4 log PFU), and MNV (approximately 7.6 log PFU). The bacteria and virus mixtures were incubated at RT for up to 2 h. Viruses without exposure to bacteria served as positive controls, which were held for the same incubation time as experimental groups. In addition, PBS alone was used a negative control. Following incubation, each mixture was filtered by a 0.22-µm mixed cellulose ester membrane filter (MilliporeSigma) and quantified by plaque assay to determine the unbound virus.

#### Lysed Bacteria and Virus

One hundred microliters of TuV (5.5 log PFU) or MNV-1 (5.6 log PFU) were mixed with 100-µL lysed *B. cereus* or *E. cloacae* in sterile 1.5-mL microcentrifuge tubes. The mixture was incubated at RT for up to 2 h (samples were collected at 0, 10, 30, 60, and 120 min). After incubation, the virus infectivity was determined by plaque assay. Virus incubated with PBS served as a control.

#### BEC and Virus

Six BECs were analyzed, including *E. cloacae* EPS and *B. cereus* EPS, *E. cloacae* LPS, biotinylated LPS from *E. coli* O111:B4 (MilliporeSigma, Burlington, MA), *P. ananatis* LPS, and *B. cereus* PG. For each sample, 100 µL of TuV (5.5 log PFU), MNV-1 (5.2 log PFU), or AiV (5.5 log PFU) was mixed with 100 µL of BEC, resulting in a total 200-µL mixture. The mixture was incubated at RT for up to 2 h to facilitate virus binding, and samples were collected at 0, 10, 30, 60, and 120 min. Following incubation, virus infectivity was quantified by plaque assay for each tested virus. BEC binding assays were compared to PBS treatment at each tested time.

#### Transmission Electron Microscopy (TEM)

TEM imaging of bacteria–virus association assay was performed after virus incubation with bacteria for 1 h. After the bacteria–virus mixture was pelleted as described above, the pellet was washed and resuspended in 400-µL DEPC-treated water. The prepared bacteria–virus suspensions were placed on collodion membrane-coated copper grids and stained with 2% phosphotungstic acid hydrate (PTA, pH 7.2) for 2 min. Virus–bacteria associations were visualized using a JEOL JEM-1011 (JEOL USA, Inc., Peabody, MA).

### Statistical Analysis

A split-plot design was used to compare the association and infectivity of viruses incubated with bacterial whole cell and components. All experiments were conducted twice, each with technical duplicates. Statistical analyses were performed with log_10_-transformed virus titers. To account for the potential random effect, the data were fitted to a mixed effect model and then analyzed using ANOVA to assess association between virus and whole cell bacteria, as well as the effects of lysed bacteria and BECs on virus infectivity over time. Tukey’s tests were performed for post hoc analysis with significant level of 0.05. Statistical analyses were carried out in R version 4.4.1 (http://www.R-project.org).

## Results

### Association of Viruses with Whole Cell Bacteria

The virus–bacteria associations were analyzed by incubating each virus with bacterium at RT (Table 1). Both HuNoV surrogates and AiV readily associated with all bacterial species within 10 min at RT with complete virus association at 60 min. The limit of detection of unbound virus (%) for TuV, MNV, and AiV were 2.0E−5, 1.6E−6, and 2.8E−6, respectively. The association maintained during the 120-min observation time (data not shown). A significantly higher % of unbound virus was observed for TuV (29 ± 15%) compared to both AiV (3.7 ± 2.0%) and MNV (3.0 ± 3.5%) at 10 min (*p* < 0.0001). At 30 min, % unbound MNV (0.3 ± 0.2%) was significantly lower (*p* < 0.0019) than both TuV (1.5 ± 1.5%) and AiV (1.9 ± 1.2%). Moreover, differences in binding activity of viruses to specific bacteria strains were observed. For instance, % unbound TuV to *E. sibiricum*—a gram-positive lettuce isolate—was significantly higher than other bacteria at specified time points. Also, lower % binding to two gram-positive bacteria *B. cereus* and *E. sibiricum* was observed for all three viruses.

**Table 1:** Association of viruses with whole cell bacteria in suspension at RT over time

Virus	Time	Percentage of unbound virus (%)*					
		*B. cereus*	*E. cloacae*	*E. sibiricum*	*P. ananatis*	*P. agglomerans*	*Pseudomonas sp.*
	10	4.28 ± 0.19^b^	1.77 ± 0.14^ab^	6.90 ± 0.96^b^	1.78 ± 0.001^a^	3.58 ± 0.18^a^	3.45 ± 0.98^ab^
AiV	30	2.54 ± 0.04^a^	0.50 ± 0.06^a^	4.00 ± 0.00^a^	1.41 ± 0.09^a^	1.42 ± 0.02^a^	1.98 ± 0.12^a^
	60	7.7E−05 ± 4.24E−06^a^	6.5E−05 ± 4.2E−06^a^	8.5E−05 ± 1.5E−05^a^	4.8E−05 ± 2.8E−06^a^	4.8E−05 ± 1.4E−06^a^	4.6E−05 ± 7.1E−06^a^
	10	7.54 ± 1.73^b^	0.64 ± 0.09^ab^	7.00 ± 0.51^b^	0.70 ± 0.11^a^	0.68 ± 0.04^a^	0.43 ± 0.02^ab^
MNV	30	0.53 ± 0.03^a^	0.07 ± 0.01^a^	0.48 ± 0.10^a^	0.43 ± 0.13^a^	0.32 ± 0.03^a^	0.03 ± 0.01^a^
	60	7.0E−05 ± 9.2E−06^a^	4.1E−05 ± 1.3E−05^a^	5.9E−05 ± 1.4E−05^a^	3.0E−05 ± 1.1E−05^a^	4.1E−05 ± 1.3E−05^a^	1.3E−05 ± 2.1E−06^a^
	10	37.50 ± 5.03^b^	21.75 ± 4.21^ab^	44.80 ± 17.21^b^	5.29 ± 1.31^a^	9.73 ± 0.39^a^	24.48 ± 0.53^ab^
TuV	30	3.99 ± 0.15^a^	0.57 ± 0.002^a^	2.73 ± 0.63^a^	0.73 ± 0.19^a^	0.30 ± 0.09^a^	0.34 ± 0.06^a^
	60	1.1E−03 ± 6.1E−04^a^	3.0E−04 ± 6.0E−05^a^	5.9E−04 ± 7.6E−05^a^	1.9E−04 ± 1.0E−04^a^	3.7E−04 ± 2.0E−04^a^	2.0E−04 ± 3.5E−05^a^

*The percentage of unbound virus are shown as mean ± SD with significant letter notations. Different letters in the table indicate significant differences. Groups sharing the same letters indicate no significant differences (*p* < 0.05)

### TEM Observation of Virus and Bacterial Whole Cell Interaction

**Fig. 1:**
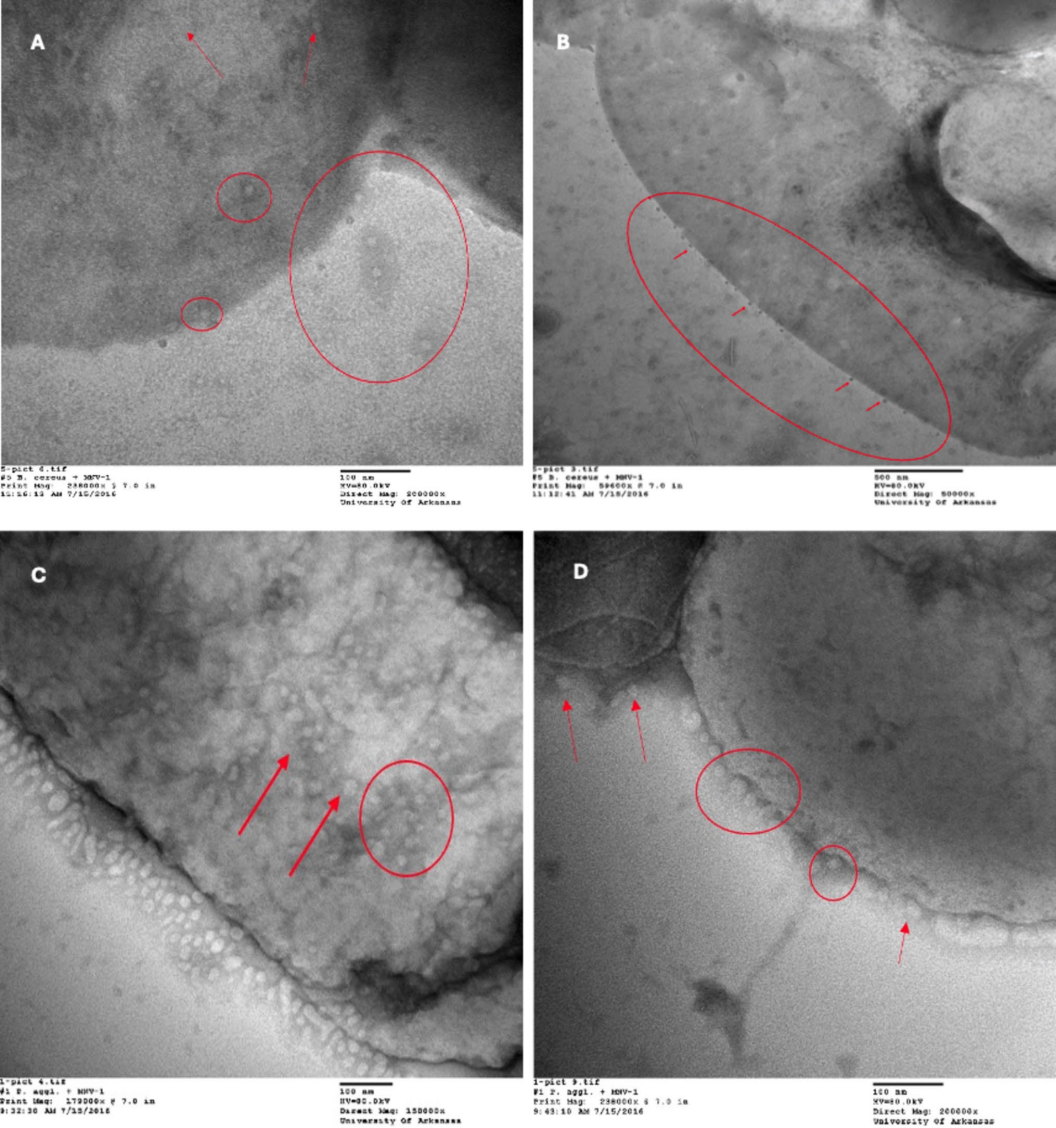
TEM images showing associations of MNV and bacterial whole cell after 60-min RT incubation. The virus–bacteria pairs MNV-1 with *B. cereus* (**A, B**) and MNV with *P. agglomerans* (**C, D**) are presented. Red circle and arrows highlight virus and bacteria association sites (Color figure online)

Select co-incubated virus and bacteria (MNV + *B. cereus*, MNV + *P. agglomerans*) were visualized by TEM (Fig. 1). After 60-min co-incubation, the majority of MNV have associated with bacteria cell surfaces, while some particles remained in suspension. Based on observed images, MNV were more densely distributed on cell surface of *P. agglomerans* than *B. cereus*.

### Impact of Co-incubation with BECs on Viral Infectivity

**Fig. 2:**
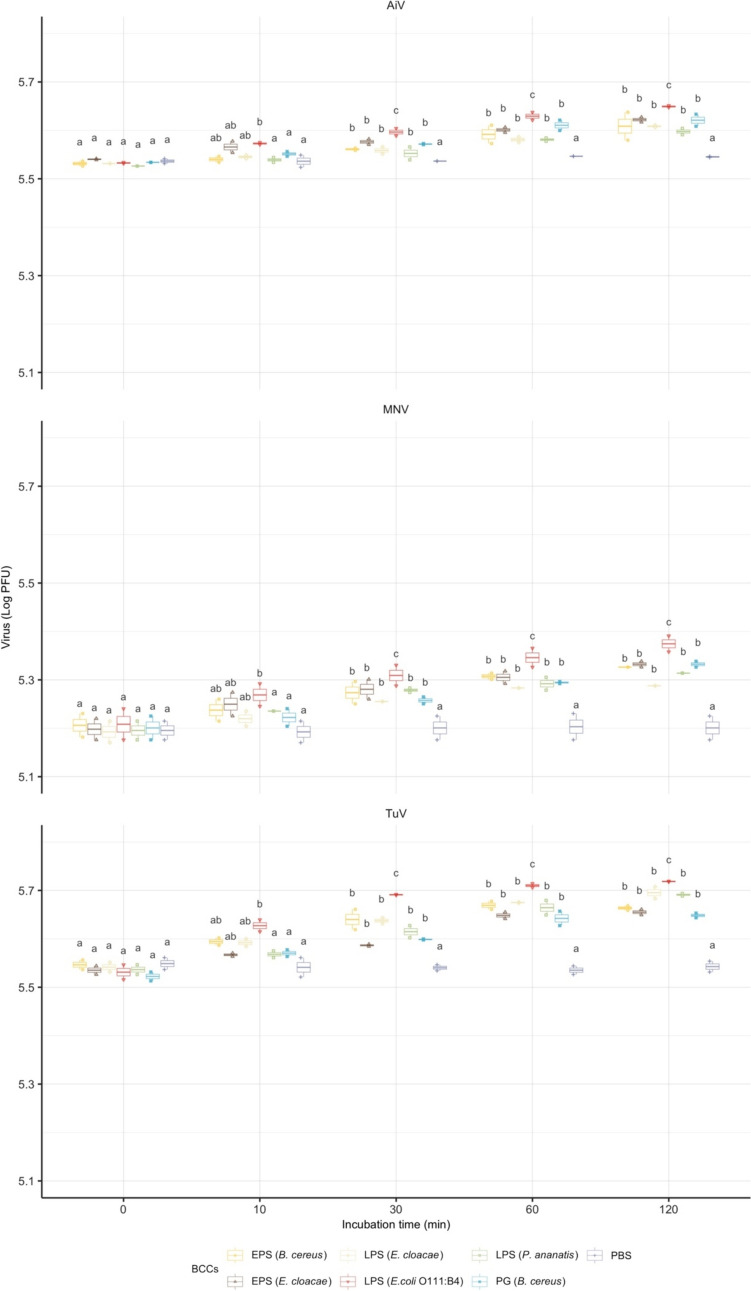
The effect of bacterial cell wall components from selected strains on infectivity of AiV, MNV, and TuV over time. The *x*- and *y*-axis represent co-incubation time and total virus recovered per sample (log PFU). The virus numbers are shown as box plot with overlaid jittered points representing the experimental means. The letters above boxes indicate statistically significant differences among treatment levels within each virus type. Groups sharing the same letters are considered no significant differences (*p* < 0.05) (Color figure online)

Given the faster association rate of viruses with gram-negative bacteria, the major differences in cell surface structure between gram-positive and gram-negative bacteria, i.e., PG and LPS, as well as EPS were further investigated. The BECs from four strains were extracted and co-incubated with viruses to observe their impact on viral infectivity over time (Fig. 2). Overall, BECs significantly increased viral infectivity compared to PBS control as determined by plaque assay (*p* < 0.0001). The enhancing effects of BECs on three viruses showed similar patterns. From 10 to 120 min, the increases in infectivity by LPS (*E. coli* O111:B4) were significantly greater than the rest BECs within each virus types (*p* < 0.05). At 120 min, the max increases on viral infectivity were up to 0.19, 0.18, 0.10 log PFU for TuV, MNV, and AiV, respectively. Regardless of the LPS of *E. coli*, virus infectivity after 30 min significantly increased in response to the other five BECs compared to 0 min. This enhancement slowly increased from 30 to 120 min while statistically insignificant.

### Impact of Co-incubation with Bacterial Whole Cell Lysates on Viral Infectivity

**Fig. 3:**
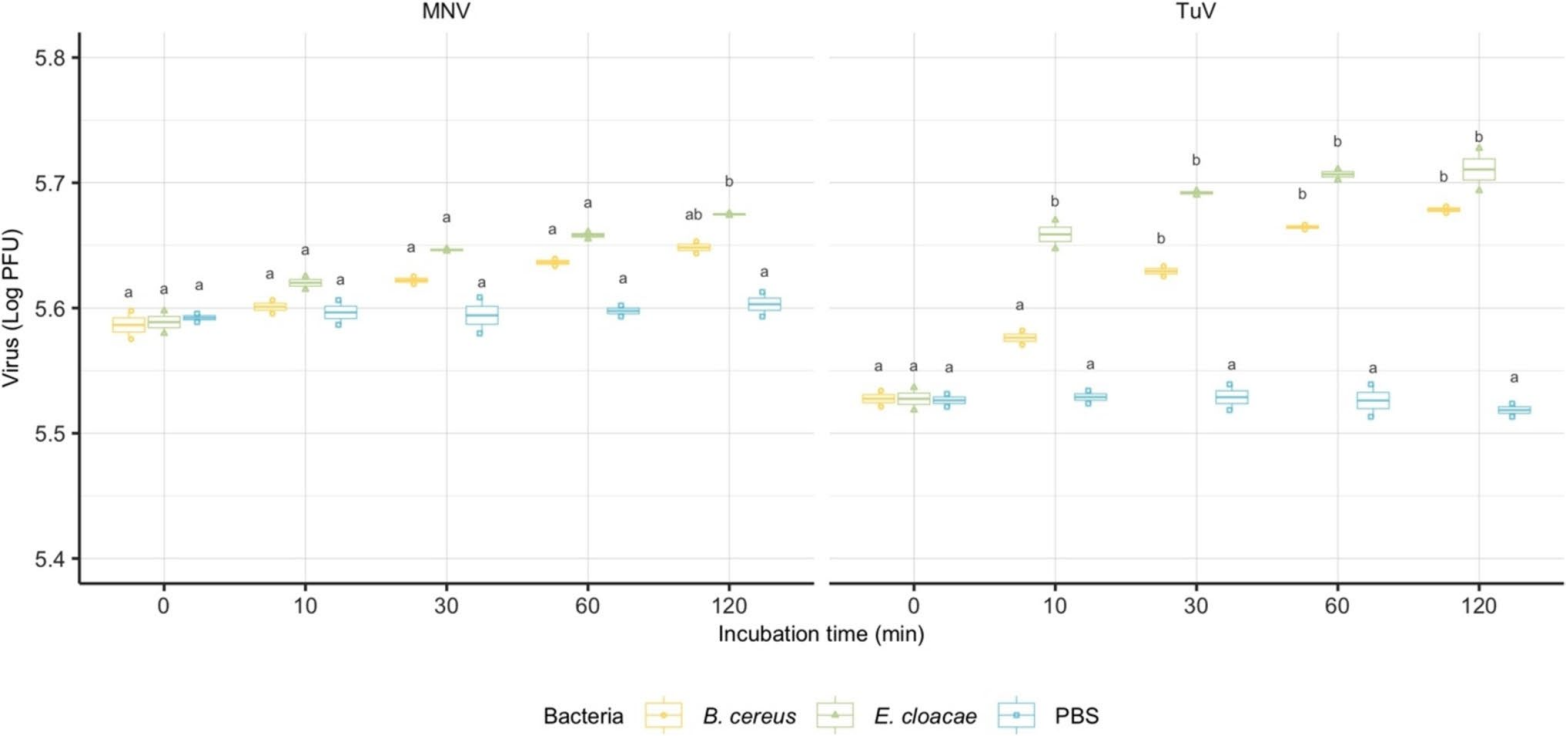
Virus infectivity over time during co-incubation with whole cell lysate of *B. cereus* and *E. cloacae*. Virus types are indicated at the top. The *x*-axis represents incubation time, while *y*-axis represents total recovered viruses in each sample. The data are presented as box plot with overlaid jittered points representing the experimental means. The letters above boxes indicate statistically significant differences among treatment levels within each virus type. Groups sharing the same letters indicates no significant differences (*p* < 0.05) (Color figure online)

To understand the effect of bacterial whole cell lysate on virus infectivity, *B. cereus* and *E. cloacae* were co-incubated with TV and MNV in pairs at RT for up to 120 min (Fig. 3). The viral infectivity was enhanced by both bacterial cell lysates as compared to PBS control. Though the differences were statistically insignificant, *E. cloacae* consistently contributed to greater increases than *B. cereus* at all time points for both viruses. However, only the increases in TuV were statistically significant, indicating the enhancing effect of cell lysate was virus specific. With the co-incubation of *E. cloacae* lysate, the infectivity of TuV increased by 0.2 logs at 120 min compared to PBS (*p* = 0.0055).

## Discussion

In recent years, numerous studies reported the interactions between enteric viruses and a range of bacteria from the human gut, food, and environment (Jones et al., 2014; Waldman et al., 2017). Bacteria can act as co-factors in virus infection, agents for virus bioaccumulation, and shelter against various stresses (Li et al., 2015; Liu et al., 2020; Neu & Mainou, 2020). However, the mechanisms of virus–bacteria interactions are complex due to the differences in bacterial surface structure and receptor specificity of viruses. This research characterized the association between enteric viruses and bacteria over time and illustrated the effect of BEC and bacteria whole cell lysate on virus infectivity.

During co-incubation at RT, viruses used in the present study associated with bacteria at different rates and once occurred, the associations maintained throughout the 2 h observation without decrease. A previous study on the interaction between MNV and 11 commensal bacteria also reported the immediate binding between virus and bacteria (Madrigal et al., 2020). However, the binding started declining after 30 min with varying rates depending on bacteria type ultimately reducing by 2 log genome copies for *Bacteroides dorei* and *Lactobacillus acidophilus* after 24 h. In contrast, another study investigated TuV interaction with 11 bacteria from a lab culture collection, human intestine, food processing equipment, and lettuce by incubating at 22 or 37 °C for 2 h and observed the absence of direct and stable virus–bacteria associations (Shearer & Kniel, 2020). However, the present study cannot directly compare with the findings of Shearer and Kniel (2020) due to the differences in experimental condition, bacteria type, incubation temperature, and detection methods. Additionally, the bacteria growth medium and growth phase could affect bacterial surface structure and thus alter the virus binding (Long et al., 2022).

The virus and bacterial whole cell association exhibited in a time-dependent pattern with variations according to specific virus and bacterium types. For instance, TuV showed the slowest association rate with all bacteria at 10 min compared to MNV and AiV. The discrepancy may be attributed to different ligands that viruses recognize on bacteria surfaces as well as varying binding affinities (Peters et al., 2022). As HuNoV surrogates, TuV recognizes HBGAs, a primary HuNoV receptor, as well as sialic acids, while MNV binds to sialic acids residues of ganglioside and glycoprotein CD300lf (Karst & Wobus, 2015; Orchard et al., 2016; Tan et al., 2015; Zhang et al., 2015). The receptors of AiV for host cell attachment have not been identified; however, its infection of Vero cells can be inhibited by synthetic peptide derived from polyproline-II helix thus indicating potential involvement in receptor interaction (Zhu et al., 2016). The presence of HBGA-like moieties on the surface of various bacteria have been shown to facilitate the binding of HuNoV and its surrogates to bacteria (Madrigal et al., 2020; Miura et al., 2013). Previous studies have reported the presence of HBGA and sialic acid sugars on bacteria surfaces, but other unknown viral binding ligands might also exist (Miura et al., 2013; Tiralongo, 2010). Moreover, the present study observed lower viral binding to gram-positive bacteria as compared to gram-negative bacteria. Previous studies on gut bacteria have shown that HBGA profiles among gram-negative Enterobacteriaceae were similar, differing from gram-positive bacteria (Almand et al., 2019). However, since MNV and AiV do not specifically recognize HBGAs for cell entry, other surface components responsible for the difference should be investigated in future research.

Given the observed effect of gram type on virus binding, the major BECs LPS, EPS, and PG were further studied for their roles in virus infection. Pronounced virus infectivity was observed following incubation with all BECs compared to the PBS control and the effect increased with extended incubation time. The envelope components of enteric bacteria are known to enhance the attachment, replication, and infection of enteric viruses (Berger & Mainou, 2018). The incubation with PG and LPS at RT for 2 h prevented reovirus from losing infectivity by nearly 50% compared with PBS (Berger et al., 2017). Robinson et al. (2014) focused on the mechanism of bacteria enhancing poliovirus infectivity and revealed that the bacterial LPS containing N-acetylglucosamine stabilized virus by preventing premature RNA release. In the present study, the LPS of *E. coli* O111:B4 improved virus infectivity more than LPS of *E. cloacae* and *P. ananatis*, which might be due to the higher purity of commercial LPS enabling more efficient virus binding. Also, the structural differences of LPS from different strains could affect the interaction with viruses (Farhana & Khan, 2024).

Previous research has also reported that bacteria surface components increase the virus resistance against various inactivation treatments (Berger et al., 2017; Deng et al., 2019). Under heat treatment at 46 °C for 4 h, the infectivity of MNV was significantly preserved by lipoteichoic acid from gram-positive bacteria and LPS from gram-negative bacteria as compared to PBS control (Budicini & Pfeiffer, 2022). Similarly, the co-incubation with LPS stabilized six picornaviruses including AiV under bleach treatment, maintaining the infectivity of 100% of input AiV (Aguilera et al., 2019). Another study pre-incubated TuV with LPS (*E. coli* O111:B4) and PG (*B. cereus*) at 37 °C for 2 h and evaluated the subsequent virus resistance against thermal and chemical inactivation (Shearer & Kniel, 2020). The PG increased the survival of TuV by more than 1.7 and 0.14 log PFU/ml under 60 °C and 200-ppm chlorine treatments, respectively. The co-incubation with EPS of a leafy green bacterial isolate contributed to significantly less reduction in infectious viruses of TuV and MNV on lettuce leaves at RT as well as when viruses were treated by 30 and 300 ppm chlorine (Liao et al., 2023). The interaction of viruses with EPS has been extensively reported as the presence of HBGA-like substances has been found in the EPS of bacteria isolates from human intestines, leafy greens, and seafood (Liao et al., 2023; Yu et al., 2023). Moreover, HBGA-expressing bacteria have also been reported to protect HuNoV under UV irradiation both in suspension and on lettuce leaf surface (Xu et al., 2021).

The whole cell lysates of *B. cereus* and *E. cloacae*, representing each gram type, also increased the infectivity of MNV and TuV in the present study. Although the increase in infectivity was similar to that observed with individual BEC, the results were not comparable because the concentrations of cell components may be different in the two experiments. The enhancing effect of *E. cloacae* on virus infectivity was greater than that of *B. cereus*, while similar distinctions were not observed for extracted BECs from two bacteria. Further study should be carried out to determine whether the gram type of bacteria or other key components contributed to the difference in infectivity.

Lastly, there are several limitations and unanswered questions in this study. The virus associations were observed in all six bacteria strains, but only three strains were selected for the subsequent BEC testing due to constraint in time and effort. Also, the mechanisms of increased virus infection by BEC were not further studied in relation to the structures and functions of BECs. In the present study, EPS promoted virus infection, while previous studies have reported antiviral activity of EPS produced by lactic acid bacteria against feline calicivirus, influenza virus, and human adenovirus (Biliavska et al., 2019; Noda et al., 2021). The examination of the biochemical characteristics of BECs may assist in understanding their roles in virus infection.

Overall, this study demonstrated that enteric viruses can associate with bacteria—both lab and fresh produce isolates—with higher binding efficiency to gram-negative bacteria. The further investigation on extracted BEC and whole cell lysates of bacteria all showed enhancement of virus infection, especially for TuV. These findings highlighted the significant roles that bacteria can play in virus infection and provided insights for future studies on virus survival and life cycles in the host. In addition, characterizing virus–bacteria interactions will support the development of control strategies for the transmission of enteric viruses in food and the environment.

## Data Availability

No datasets were generated or analyzed during the current study.
